# Global Proteomic Profiling of Pediatric AML: A Pilot Study

**DOI:** 10.3390/cancers13133161

**Published:** 2021-06-24

**Authors:** Nam H. K. Nguyen, Huiyun Wu, Haiyan Tan, Junmin Peng, Jeffrey E. Rubnitz, Xueyuan Cao, Stanley Pounds, Jatinder K. Lamba

**Affiliations:** 1 Department of Pharmacotherapy and Translational Research, Center for Pharmacogenomics, College of Pharmacy, University of Florida, Gainesville, FL 32610, USA; namnguyen@ufl.edu; 2 Department of Biostatistics, St. Jude Children’s Research Hospital, Memphis, TN 38105, USA; huiyun.wu@stjude.org (H.W.); stanley.pounds@stjude.org (S.P.); 3 Center for Proteomics and Metabolomics, St. Jude Children’s Research Hospital, Memphis, TN 38105, USA; haiyan.tan@stjude.org (H.T.); junmin.peng@stjude.org (J.P.); 4 Department of Structural Biology, St. Jude Children’s Research Hospital, Memphis, TN 38105, USA; 5 Department of Developmental Neurobiology, St. Jude Children’s Research Hospital, Memphis, TN 38105, USA; 6 Department of Oncology, St. Jude Children’s Research Hospital, Memphis, TN 38105, USA; jeffrey.rubnitz@stjude.org; 7 College of Nursing, University of Tennessee Health Science Center, Memphis, TN 38163, USA; xcao12@uthsc.edu; 8 UF Health Cancer Center, University of Florida, Gainesville, FL 32610, USA

**Keywords:** pediatrics acute myeloid leukemia, leukemic cells, untargeted labeled proteomics, tandem mass tag, transcriptomics

## Abstract

**Simple Summary:**

The second most common childhood leukemia, acute myeloid leukemia (AML), is a heterogeneous disease with a poor prognosis. In order to improve outcomes, efforts to understand the genomic/transcriptomic landscape of AML have been performed. However, there is a significant gap in our understanding of leukemic cell proteomics. In comparison to the proteome, the transcriptome alone cannot adequately represent the biological functions within cells and it is often not a target for immediate drug development. In the current study, we investigated cytogenetic differences at the proteomic level and sought potential predictive biomarkers and druggable proteins.

**Abstract:**

Acute Myeloid Leukemia (AML) is a heterogeneous disease with several recurrent cytogenetic abnormalities. Despite genomics and transcriptomics profiling efforts to understand AML’s heterogeneity, studies focused on the proteomic profiles associated with pediatric AML cytogenetic features remain limited. Furthermore, the majority of biological functions within cells are operated by proteins (i.e., enzymes) and most drugs target the proteome rather than the genome or transcriptome, thus, highlighting the significance of studying proteomics. Here, we present our results from a pilot study investigating global proteomic profiles of leukemic cells obtained at diagnosis from 16 pediatric AML patients using a robust TMT-LC/LC-MS/MS platform. The proteome profiles were compared among patients with or without core binding factor (CBF) translocation indicated by a t(8;21) or inv(16) cytogenetic abnormality, minimal residual disease status at the end of the first cycle of chemotherapy (MRD1), and in vitro chemosensitivity of leukemic cells to cytarabine (Ara-C LC50). Our results established proteomic differences between CBF and non-CBF AML subtypes, providing insights to AML subtypes physiology, and identified potential druggable proteome targets such as *THY1 (CD90)*, *NEBL*, *CTSF*, *COL2A1*, *CAT*, *MGLL (MAGL)*, *MACROH2A2*, *CLIP2* (isoform 1 and 2), *ANPEP (CD13)*, *MMP14*, and *AK5.*

## 1. Introduction

Acute myeloid leukemia (AML) is the second most common childhood leukemia with five-year survival rates of approximately 60% [1]. AML is an aggressive and heterogenous malignancy characterized by clonal disorder of hematopoietic stem or progenitor cells, resulting in the accumulation of deformed, immature, and nonfunctional myeloid cells in bone marrow and blood. Genetic abnormalities play a critical role in the pathogenesis of AML. Specifically, core binding factor (CBF) AML with the presence of t(8;21)(q22;q22) or inv(16)(p13q22)/t(16;16)(p13;q22) cytogenetic abnormalities, resulting in *RUNX1/RUNX1T1* and *CBFβ/MYH11* fusions, respectively, account for approximately 30% of pediatric AML [2]. The CBF AML subtype is associated with a better prognosis, while outcomes are inferior in patients with non-CBF AML [3].

Recent advances in next-generation sequencing technologies have significantly expanded researchers’ understanding of pediatric AML heterogeneity. However, most of these studies have focused on differential transcriptomic profiles [4,5,6]. Whether transcriptome features translate to the protein level remains to be investigated. Additionally, uncovering genetic mutations and abnormal gene expressions is less likely to lead to the immediate therapeutic option, as most druggable targets are proteins rather than genes/transcripts. Therefore, the characterization of the proteomic landscape is critical. Although, as of April 2021, an ongoing effort by the National Cancer Institute in Proteomics Data Commons database has data concerning ~15,000 proteins in 11 different cancer primary sites, there are no data for AML (https://pdc.cancer.gov/pdc/ accessed on 13 May 2021). Currently, only a handful of studies have investigated proteome profiles in AML, none of which have focused on global untargeted proteomic profiling in pediatric AML [7,8,9,10,11,12,13]. It is well established that the molecular landscape of pediatric AML is distinct from adult AML with age-specific mutational interactions, highlighting the need for proteomic investigation in the pediatric population in order to further characterize the heterogeneity and develop treatments with age-specific targeted therapies for pediatric AML [14].

The objective of this pilot study is to examine proteomic features and biological differences in CBF and non-CBF pediatric AML, as well as to uncover proteomic markers that are associated with the therapeutic responses through minimal residual disease post first induction (MRD1) and in vitro leukemic cell cytarabine chemosensitivity (Ara-C LC50). Our exploratory objective was to estimate the correlation between proteome and transcriptome from available transcriptome data.

## 2. Materials and Methods

### 2.1. Study Cohort, Proteomic and Transcriptomic Expression Profiling

The study cohort included bone marrow leukemic blasts obtained at diagnosis from 16 pediatric AML patients treated on the multicenter AML02 clinical trial (NCT00136084). Details of study design and clinical outcomes have been previously described [15]. Summarily, patients were randomized to receive high (3 g/m^2^, given every 12 h on days 1, 3, and 5) or low dose (100 mg/m^2^ given every 12 h on days 1–10) cytarabine with daunorubicin and etoposide as a first course of chemotherapy, and treatments were subsequently tailored to response and risk classification. Cytogenetic analysis, *FLT3* internal tandem duplications (ITD), and *FLT3* point mutations were identified by conventional cytogenetics and/or PCR. CBF AML included t(8;21)(q22;q22) or inv(16)(p13q22)/t(16;16)(p13;q22), while non-CBF AML was defined as the absence of t(8:21) or inv(16). MRD1 was measured by flow cytometry and was defined as positive with the presence of one or more leukemic cells per 1000 mononuclear bone marrow cells (i.e., ≥0.1%). Ara-C LC50 (the concentration of cytarabine required for 50% leukemic cell kill) values were determined from leukemic cells treated with varying concentrations (ranging from 0.002 to 2.5 ng/μL) of cytarabine as described previously [16,17]. Furthermore, the 16 pediatric AML were randomly selected from AML02 patients with bone marrow aspirate available, balancing CBF vs. non-CBF, MRD1 status, and Ara-C LC50 level. The St. Jude Institutional Review Board approved this study, and consent was secured from both parents/guardians and the consenting individuals.

Proteomic profiling was performed using an untargeted global approach a with 16-plex isobaric tandem mass tag (TMT) labeling reaction, two-dimensional reversed-phase liquid chromatography (LC/LC) fractionation, and tandem mass spectrometry (MS/MS), followed by computational data processing. A complete detail of the TMT-LC/LC-MS/MS procedure has been previously described [18]. Briefly, 20 μg of extracted proteins from bone marrow samples with appropriately 1 million leukemic blasts were digested and labeled with 16 different TMT tags. Samples from 16 channels were then pooled equally into a mixture, and the mixture was fractionated into 60 fractions by basic pH reverse-phase LC. Each fraction was further analyzed by acidic pH reverse phase nanoscale LC and high-resolution mass spectrometry (Q Exactive HF, Thermo Fisher Scientific, Waltham, MA, USA). The raw data was processed by in-house JUMP (Jumbo Mass Spectrometry-based Proteomics Tool) software (https://github.com/JUMPSuite/JUMP accessed on 13 May 2021, Peng Lab, St. Jude Children’s Research Hospital, Memphis, TN, USA), and spectra were searched against the Uniport human database with proteins identified at a 1% false discovery rate (FDR) [19,20]. For quantification, preprocessed peptides/proteins were measured by the TMT reporter ions [21]. Data were further normalized and transformed by variance stabilizing transformation (vsn) in order to reduce intragroup variation prior to any downstream analysis [22,23]. Vsn was observed to perform the best in reducing the intragroup variation compared to other normalization methods including log2, median, mean, and quantile for TMT proteomic quantification normalization [24]. Moreover, the vsn normalization performed a transformation similar to the log transformation and required the input to be untransformed [22].

Gene expression profiling of bone marrow leukemic blasts at diagnosis was obtained using the GeneChip^®^ Human Genome U133A [Affymetrix, Santa Clara, CA, USA] as described previously [25]. The MAS 5.0 algorithm was used to achieve normalized gene expression signals. Vsn was also applied to improve the low-intensity expression estimates and ensure variance stabilization and calibration in microarray data [22].

### 2.2. Differential Expression Analysis of Proteomics Data and Integrative Analysis

Differential protein expression analysis was analyzed in R studio software (version 4.0.3, R Foundation for Statistical Computing, Vienna, Austria) with Bioconductor packages. Normalized proteomic data for each patient sample were grouped for categorical analysis in three comparisons: (1) CBF status (CBF vs. non-CBF AML); (2) MRD1 status (positive vs. negative); and (3) Ara-C LC50 status (high vs. low by LC50 values median). Herein, differential protein expression for each comparison was performed using rank-sum analysis for unpaired cases via the “RankProd 2.0” package, a non-parametric method for identifying differentially expressed omics datasets [26]. A threshold for significant differentially expressed proteins (DEPs) was set at FDR < 0.05 and |Log2FC| > 1 for the differential comparison unless otherwise indicated. Hierarchical clustering of significant DEPs was evaluated for each comparison, and heatmaps were plotted with scaled relative expression among samples. Since CBF status was a comparison between CBF vs. non-CBF AML, proteins with a positive Log2FC were classified as upregulated in CBF AML, whereas proteins with a negative Log2FC as upregulated in non-CBF AML.

Integrative analysis among three differential comparisons (CBF, MRD1, and LC50 status) were executed in order to identify proteins with significant prognoses and/or potential drug targets.

### 2.3. Protein–Protein Interaction (PPI), Pathway Analysis, and Gene Set Enrichment Pathway (GSEA) of CBF Status Comparison

To investigate the biological functions and processes which are different between CBF and non-CBF AML, we utilized various publicly accessible databases. First, STRING database version 11.0 was utilized to construct an entire PPI network of significant DEPs [27], including interactions from experiments, databases, co-expression, co-occurrence, gene fusion, homology, neighborhood, and text mining. Minimum required interactions were set at a default score of 0.400 with FDR < 0.05. Next, Cytoscape open-source software (https://cytoscape.org/ accessed on 13 May 2021, version 3.8.2,) was employed to visualize the PPI network relationship between significant DEPs with exclusion of singleton interactions [28].

Subsequently, we used ClueGO version 2.5.7, a Cytoscape plug-in, to visualize the non-redundant biological terms utilizing three signature databases (Gene Ontology [GO] with three major sub-ontologies with Biological Process [BP], Cellular Component [CC], and Molecular Function [MF] [29]; Reactome [30]; and Kyoto Encyclopedia of Genes and Genomes [KEGG] [31]) for large clusters of genes in a functionally grouped network [32]. The grouping network was set at a kappa score threshold of 0.4 with FDR < 0.05. We then performed the Pathview analysis of the most common significant abundance grouped network with KEGG pathway on significant DEPs regarding CBF status [33].

GSEA was achieved using pre-ranked scores from proteins, with Log2FC indicating the direction of enrichment and *p*-values indicating the degree of significance (pre-rank scores were calculated by the sign of Log2FC multiplied by −log10 of *p*-values) [34]. The default-weighted enrichment-based statistic was adapted to conduct 10,000 permutations with a minimum and maximum criterion for selecting gene/protein sets from database collections were 10 and 500 gene/protein, respectively. FDR < 0.05 was considered significantly enriched. The “ClusterProfiler” R package [35] was used to analyze and visualize the GSEA in GO, Reactome, and KEGG databases.

### 2.4. Correlation Analysis between Proteomics and Transcriptomics Data

Within-gene pairwise correlations of observed transcripts and proteins expression were conducted for the 15 samples with paired gene expression profiles. The Spearman’s rank correlation coefficient was calculated between proteome and transcriptome with a matched gene symbol among 15 samples. A correlation estimate percentage was then computed for negative correlation, positive correlation, and significant correlation (*p*-value < 0.05) at a global level and on significant DEPs of three differential comparisons. For genes that have multiple protein–transcript combinations (i.e., one protein with multiple transcript probes for a common gene and vice versa), only protein–transcript pairs with the lowest *p*-value for that gene were selected in the final correlation estimation.

## 3. Results

### 3.1. Study Cohort and Proteomic Profiling

Sixteen pediatric AML patients treated on the AML02 clinical trial were randomly selected and included in this study. The median age was 11.4 years with a range of 3 to 21.2 years; 88% were white; the median white blood cell count was 33 with a range of 6 to 351 × 10^9^/L; and 50% of patients were positive for MRD1 (Table 1). Among these patients, six had CBF and ten had non-CBF AML, eight were MRD1-positive, and eight were MRD1-negative. In vitro Ara-C LC50 data on diagnostic leukemic cells was available from 13 patients, and the median Ara-C LC50 was 0.29 with a range of 0.1 to 1.79 ng/μL. Thus, seven patients were classified into the LC50-high and six into the LC50-low group.

Global untargeted proteomic profiling with 16-plex TMT-LC/LC-MS/MS identified and quantified a total of 10,634 proteins for each sample (FDR < 0.01). Following additional quality control, a total of 9800 annotated proteins per sample were included for downstream analysis.

### 3.2. Proteomic Profiling of CBF Compared to Non-CBF AML Patients and Functional Analysis

**Figure 1:**
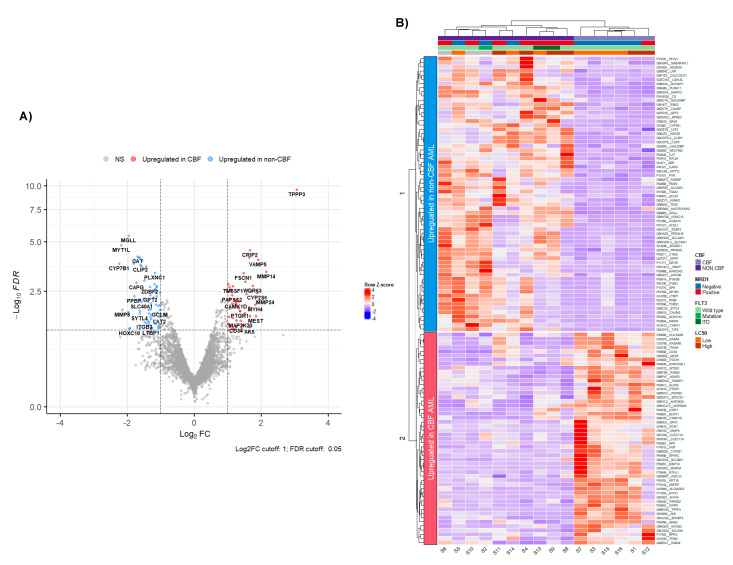
Leukemic cell proteomic profiles comparing CBF AML with non-CBF AML patients: (**A**) Volcano plot illustrating differentially regulated protein expression in patients with CBF AML (*n* = 6) compared with non-CBF AML (*n* = 10). Differential protein expression revealed a total of 51 upregulated proteins in CBF (red) and 66 upregulated proteins in non-CBF (blue) (FDR < 0.05, |Log2FC| > 1); (**B**) Heatmap with hierarchical clustering of the 117 differentially expressed proteins between CBF and non-CBF AML, color scaled by row Z-score indicates relative expression (high levels of expression in red and low levels of expression in blue).

Comparing proteomic profiles of CBF to non-CBF AML resulted in 117 significant DEPs (FDR < 0.05, |Log2FC| > 1) with 51 positive and 66 negative Log2FC as shown in the volcano plot (Figure 1A). Fifty-one proteins were significantly upregulated in CBF AML, and sixty-six proteins were significant upregulated in non-CBF AML. A comprehensive list of DEPs with CBF compared to non-CBF is given in Table S1. A heatmap with hierarchical clustering of 117 DEPs resulted in a successful separation of patients with CBF and non-CBF AML patients into two discrete groups, indicating a differential proteins signature for CBF vs. non-CBF AML (Figure 1B). The top 10 differential proteins included upregulated proteins *TPPP3*, *CRIP2*, *SLC9A3R2*, and *VAMP5* in CBF AML, and upregulated proteins *MGLL*, *MYT1L*, *CAT*, *TESC*, *CLIP2*, and *CS* in non-CBF AML.

**Figure 2:**
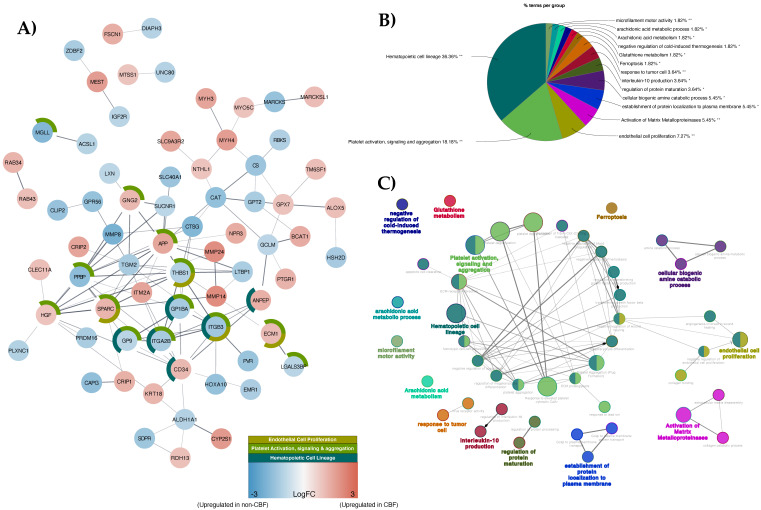
Interaction and functional analysis of 117 significant DEPs present in CBF and non-CBF AML comparison: (**A**) STRING protein–protein interaction (PPI) network of significant DEPs. Nodes represent proteins, and edges connecting nodes represent interaction. The PPI network has been simplified by concealing singletons. Red positive Log2FC nodes are upregulated proteins in CBF AML, while blue negative Log2FC nodes are upregulated proteins in non-CBF AML; (**B**) Functional grouped network enrichment by ClueGO with percent terms per group, and the most significant enriched grouped terms is hematopoietic cell lineage (* or ** indicate significant terms at the *p* < 0.05 and *p* < 0.01 statistical levels, respectively); (**C**) Functional grouped terms are nodes showing only terms with most significance (FDR < 0.05), and nodes are linked base on their kappa score level (>0.4). The node size represents the number of terms enrichment significant, and functionally related groups partially overlap.

The STRING database was used in conjunction with Cytoscape in order to construct a protein–protein interaction (PPI) network on 117 significant DEPs in order to identify significant PPIs. STRING analysis resulted in 112 nodes with more interactions than expected with at least one direct or indirect association (115 connected edges vs. 49 expected edges with an average node degree of 2.05 and PPI enrichment *p*-value = 1.33 × 10^−15^) (Figure 2A). ClueGO functional grouped network analysis among significant DEPs showed 15 significant grouped enrichment (FDR < 0.05) by the percentage of terms per group (Figure 2B). ClueGO grouped term network was further illustrated with Cytoscape, showing that the hemopoietic cell lineage grouped term is highly connected with other grouped terms including platelet activation, signaling and aggregation, and endothelial cell proliferation (Figure 2C).

**Figure 3:**
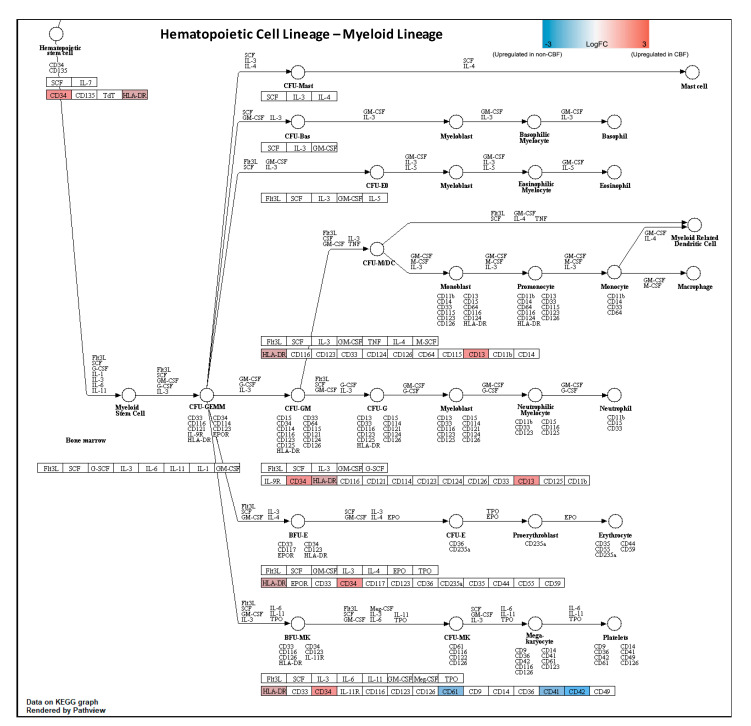
KEGG Pathview of hematopoietic cell linage of significant DEPs in CBF AML compared to non-CBF AML. Pathview analysis demonstrated majority of significant DEPs involved in the myeloid lineage differentiation. Proteins are indicated as significantly overexpressed in CBF AML (red), unchanged (gray), or overexpressed in non-CBF AML (blue). *ANPEP* also known as *CD13*; *ITGA2B* as *CD41*; *GP9* as *CD42a*; *GP1BA* as *CD42b*; and *ITGB3* as *CD61*.

Together, these findings suggested that CBF and non-CBF AML are both involved in hematopoietic cell lineage with distinct protein profiles in myeloid cell differentiation. Pathview analysis further illustrated expression levels of significant DEPs in hematopoietic cell lineage via KEGG pathway (individual KEGG term FDR = 6 × 10^−5^ and grouped KEGG term network FDR = 1.8 × 10^−9^) (Figure 3). Interestingly, *CD34* and *CD13* are significantly overexpressed in CBF AML compared to non-CBF AML, while *CD41*, *CD42*, and *CD61* are significantly overexpressed in non-CBF AML compared to CBF AML within myeloid cell differentiation.

**Figure 4:**
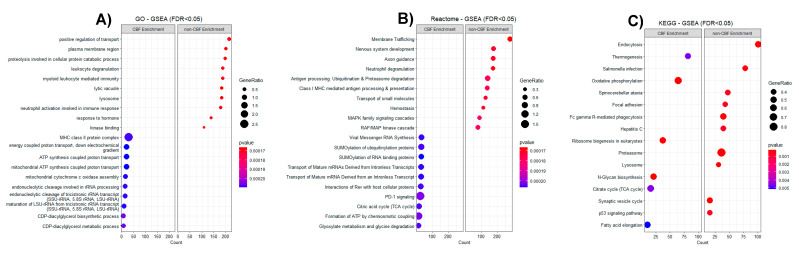
Gene Set Enrichment Analysis (GSEA) of 9800 proteins comparing CBF AML to non-CBF AML. Each dotplot showing the top 10 category enriched pathways ranked by count and FDR in CBF AML (left) or non-CBF AML (right). ‘Count’ is the number of proteins enriched in each term. ‘GeneRatio’ is the percentage of selected proteins count over the set size for a given term. (**A**) GSEA results of all GO terms including Biological Process (BP), Molecular Function (MF), and Cellular Component (CC); (**B**) GSEA results of Reactome terms; (**C**) GSEA results of KEGG terms.

Additionally, the top gene-sets enriched in CBF AML by GSEA using the GO (including BP, MF, and CC), Reactome, and KEGG databases resulted in common protein functions particularly linked to oxidative phosphorylation, RNA synthesis/processing, and multiple pathways with metabolic processes. On the other hand, gene-sets enriched in non-CBF AML revealed common functions in the regulation of transport, membrane trafficking, cellular protein catabolic process, and myeloid leukocyte mediated immunity (Figure 4). The full results of GSEA (FDR < 0.05) are included in Table S2, with positive normalized enrichment score (NES) indicating gene-sets enriched in upregulated CBF, and negative NES score indicating gene-sets enriched in upregulated non-CBF.

### 3.3. Proteomic Profiling by MRD1 Status and by In Vitro Ara-C LC50 Level

Since most proteins are druggable targets, we were interested in investigating the proteomic profile between MRD1-positive and negative patients, as well as proteomic feature profile between LC50-high vs. low. One limitation to be noted is that only 1/6 of the CBF-AML was MRD1-positive and 7/10 non-CBF AML cases were MRD1-positive. Thus, we expect some overlap in the proteomic profiles between MRD1 status and CBF status evaluations. Comparing the proteomic profile of MRD1-positive and negative status among 16 patients revealed 65 significant DEPs (*p*-value < 0.05, |Log2FC| > 1), with 17 upregulated and 48 downregulated proteins in MRD1-positive AML (Figure S1A). Hierarchical clustering of 65 significant DEPs resulted in partial separation of patients with MRD1-positive vs. negative (Figure S1B). A detailed list of the 65 DEPs can be found in Table S3. Proteins with significant overexpression within MRD1-positive group included *NT5DC3*, *CLIP2* (both isoform 1 and 2), *MACROH2A2*, *NEBL*, *MGLL (MAGL)*, *HLA-DPA1*, *HLA-DPB1*, *CAT*, *TTC41P*, *ICA1*, *TBC1D8B*, *GOLGA8K*, *THY1 (CD90)*, *HOXC10*, *HOXD10*, and *SNRPE.*

With respect to in vitro leukemic cell chemosensitivity, 13 samples had the available cytarabine concentration needed to kill 50% of leukemic cells (Ara-C LC50). Comparison of proteomic profiles between leukemic cells from patients with high vs. low Ara-C LC50 level by median value (0.29 ng/μL) identified 24 significant DEPs (*p*-value < 0.05, |Log2FC| > 1) with three upregulated and 21 downregulated proteins in high Ara-C LC50 group (Figure S2A). Hierarchical clustering of the 24 DEPs indicated the moderate separation of this group of patients (Figure S2B). Significantly overexpressed proteins for Ara-C LC50-high level are *CTSF*, *GATM*, *COL2A*. A full list of DEPs comparing Ara-C LC50-high to LC50-low groups is provided in Table S4.

### 3.4. Integrative Analysis of Three Comparison Strategies (CBF, MRD1, and Ara-C LC50)

**Figure 5:**
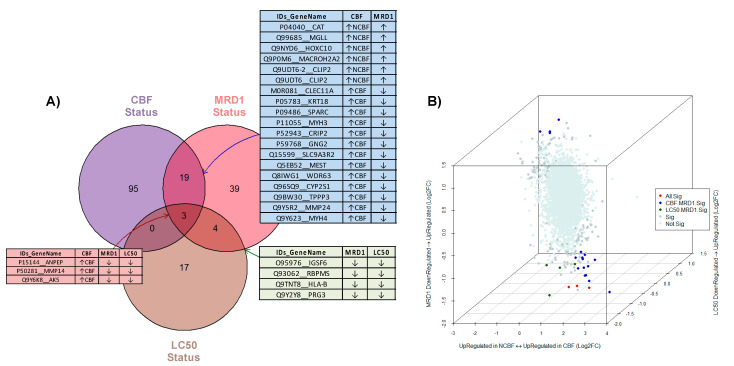
Integrative analysis of three differential comparisons (CBF, MRD1, and Ara-C LC50): (**A**) Venn diagram illustrating unique and overlapping significant DEPs between all comparisons (*n* = 177). Purple circle, CBF AML versus non-CBF AML; Pink circle, MRD1-positive versus MRD1-negative; Brown circle, Ara-C LC50-high versus Ara-C LC50-low; (**B**) 3D scatterplot with log2FC of the three comparisons: x-axis is the log2FC of CBF status with bidirectional, where positive values are upregulated in CBF and negative values are upregulated in non-CBF; y-axis is the log2FC of Ara-C LC50 (high vs. low); and z-axis is the log2FC of MRD1 (positive vs. negative). Red proteins in (**A**) table and dots are common significant proteins among three comparisons, blue proteins in (**A**) table and dots are significant proteins between CBF and MRD1 status, and green proteins in (**A**) table and dots are significant proteins between Ara-C LC50 and MRD1 status.

Integrating the 177 significant differentially expressed proteins across the three comparisons described above resulted in three common significant DEPs. Among all comparisons, 19 DEPs overlapped between CBF and MRD1 status, and four DEPs between MRD1 and Ara-C LC50 status along with expression direction for each contrast as shown in the Venn diagram in Figure 5. Detailed information of these 26 DEPs in at least two of the three comparative analyses (CBF, MRD1, and Ara-C LC50) are included in Table S5.

Given the superior prognostic nature of CBF compared to non-CBF AML subtypes, we observed the concordance of dysregulated direction between CBF status with MRD1-positive and high Ara-C LC50 level. The common significant DEPs of three comparisons included *ANPEP*, *MMP14*, and *AK5* proteins that were upregulated in CBF AML and downregulated in MRD1-positive and high Ara-C LC50 groups. Likewise, *CAT*, *MGLL*, *HOXC10*, *MACROH2A2*, and *CLIP2* (both isoform 1 and 2) were upregulated in the non-CBF and overexpressed in the MRD1-positive group. Moreover, *IGSF6*, *RBPMS*, *HLA-B*, and *PRG3* are also in concordance with downregulation in both MRD1 and Ara-C LC50 differential comparisons.

### 3.5. Correlation Analysis of Matched Proteome and Transcriptome

**Figure 6:**
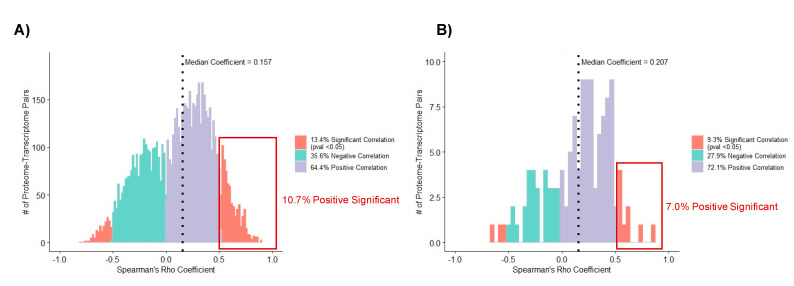
Spearman’s Rho analysis within-gene pairwise correlation of proteins to observed transcripts in matched patient leukemic samples. Teal, negative correlation; Purple, positive correlation; Orange, significant correlation (Spearman’s Rho *p*-value < 0.05) with either negative or positive correlation coefficient; Red Box, positive significant correlation. (**A**) Global correlation bar plot of available 7039 protein–gene pairs and (**B**) Correlation bar plot of 129 significant DEPs from our 3 comparative analysis with gene pairs.

Among 15 matched bone marrow samples with available transcriptomic and proteomic expression, there were 7039 common genes with 7887 proteins and 12,674 transcript probes. Within-gene pairwise Spearman’s Rho correlation with a selection of lowest *p*-value for all protein–transcript pairs, only 13.4% (*n* = 942/7039) demonstrated a significant correlation (*p*-value < 0.05) (Figure 6A) in leukemic cells from diagnosis. We also separately evaluated the 177 significant DEPs identified in our comparative analyses described above (CBF-AML vs. non-CBF-AML; MRD1-positive vs. MRD1-negative; and low vs. high Ara-C LC50) for correlation with gene expression levels from matching leukemic cells at diagnosis. Out of 177 proteins across three comparisons, only 129 protein–transcript pairs were available for correlation analysis. Our results showed significant correlation (*p* < 0.05) in only 9.3% (12/129) of the mRNA-protein pairs. (Figure 6B). Among all significant correlations, the positive significant correlation for global and significant DEPs was 10.7% and 7.0%, respectively. Furthermore, where applicable, correlation results of proteome and transcript with *p*-value and coefficient are included in all Supplementary Tables.

## 4. Discussion

Despite the growing interest in investigating proteins as functional molecules in cells, no global proteomic studies of leukemic cells in children with AML studies are currently available. In this study, we reported the proteomic profiles of pediatric bone marrow-derived AML cells at diagnosis in three differential comparison strategies: (1) core binding factor (CBF; t(8;21), inv(16)) AML vs. non-CBF AML; (2) MRD1-positive vs. MRD1-negative status; and (3) in vitro chemosensitivity of Ara-C LC50-high vs. low level. Within the first comparison, we identified a total of 117 proteins that were dysregulated between CBF AML and non-CBF AML subtypes. Interestingly, several significant DEPs have shown to be of relevance to AML at the transcriptome level such as *CRIP2*. Overexpression of *CRIP2* in CBF AML was previously reported to be highly overexpressed in leukemic stem cells (LSC) and is associated with chemotherapy resistance in AML in response to *EZH2* inactivation [36]. Moreover, *CRIP2* promotes oxidative phosphorylation and regresses glycolysis, which stabilizes metabolic features characteristic for AML stem cells [8]. Likewise, GSEA from CBF status results revealed that involvement of several metabolomics pathways such as ATP synthesis coupled proton transport, TCA cycle, and oxidative phosphorylation to biosynthesis of various metabolic precursors, suggesting the need for studying metabolic processes and profiling, especially in the CBF subset of pediatric AML. The overexpression of *SLC9A3R2* in CBF AML has previously been shown to be upregulated at relapse [37]. Among proteins relevant to non-CBF, the overexpression of *TESC* also shown to be highly expressed in *FLT3*-*ITD*(+) AML mediating sorafenib resistance [38], suggesting potential drug resistance in non-CBF AML. The overexpression of *CLIP2* (both isoform 1 and 2) in non-CBF was reported for poor progression in upregulating at the transcriptome level in large AML multi-studies [39]. This corresponded with our finding that *CLIP2* was also shown to be upregulated in an inferior clinical endpoint with MRD1-positive.

Using pathway analysis to compare CBF and non-CBF AML proteomic profiles revealed involvement in hematopoietic cell lineage development, platelet activation/signaling/aggregation, and endothelial cell proliferation. Between CBF and non-CBF AML, the KEGG pathway within Pathview showed distinct hematopoietic stem cell (HSC) profiles with unique biomarkers. Thus, this validated the observed biological functions of *RUNX1* and *CBFβ* genes in CBF AML as the transcription factor complex that is important for HSC’s emergence from an endothelial cell stage. Previously, *CBFβ-MYH11* transduction of *CD34+* cells were shown to enhance proliferation [40]. In line with these results, *CD34* protein was significantly overexpressed in CBF AML compared to non-CBF AML patients, suggesting CBF AMLs carry unique protein expressions that resemble *CD34+* progenitor cells. Fascinatingly, CBF AML protein expressions resemble the earliest identifiable megakaryocyte progenitor with the burst-forming unit, megakaryocyte (BFU-MK), while non-CBF AML has protein features of later progenitor with colony-forming unit, megakaryocyte (CFU-MK) and megakaryocyte.

The proteins that were overexpressed in non-CBF and in MRD1-positive included *CAT*, *MGLL (MAGL)*, *MACROH2A2*, and *CLIP2* (both isoform 1 and 2). Of these, *CAT* represents catalase, a key antioxidant enzyme which has been implicated in resistance to antileukemic agents such as doxorubicin [41], and *MGLL* (also known as *MAGL*) represents monoglyceride lipase, which has been shown to be overexpressed in aggressive cancers. Given its involvement in oncogenic lipid signaling, *MGLL* represents an exciting pharmacological target and there are ongoing efforts to identify the inhibitors of MAGL [42,43,44,45]. *MACROH2A2*, (also known as *H2AFY*) is a fusion partner of *MECOM* in AML [46]. Collectively, the overexpression of *CAT*, *MGLL (MAGL)*, *MACROH2A2*, and *CLIP2* (both isoform1 and isoform2) in non-CBF AML were also significantly upregulated in MRD1-positive samples, indicating possible noteworthy prognostic factors and druggable targets that would require further evaluation. Other significant DEPs among MRD1-positive and the Ara-C LC50-high level that have been shown to be relevant to AML were identified in this study. The overexpression of *THY1* (also known as *CD90*) in MRD1-positive was reported to be preferentially expressed on blast cells of high-risk AML, and *CD90+* AML has been associated with shorter patient survival [47]. Hence, *THY1* protein could carry potential prognostic significance in pediatric AML. Remarkably, the overexpression of *NEBL* in MRD1-positive was shown to be involved in MLL rearrangement, which is the most frequently identified genetic aberration in early infancy AML [48]. Among the three overexpressed proteins for high Ara-C LC50 level, *CTSF* was shown to have a function in azurophil granules and was found to be overexpressed in acute promyelocytic leukemia (APL) *FLT3-ITD* [49]. *GATM* was reported to be associated with increased reactive oxygen species within mitochondria, and found to enriched in chemo-resistant leukemic cells to Ara-C in vitro [50]. *COL2A1* was studied to be highly correlated to granulocyte–monocyte progenitor (GMP), which is closely associated with AML [51]. Together, the significant overexpression of proteins for MRD1-positive and high Ara-C LC50 level are in consistent with previous studies for poor clinical outcomes and/or AML progression, demonstrating that inhibition of these proteins could be attractive therapeutic targets.

Integrative analysis of significant DEPs among three comparisons with cytogenetics such as CBF, MRD1, and Ara-C LC50 clinical endpoints, revealed 100% concordance between cytogenetic features and clinical endpoints in our results. This suggested that the difference in prognosis between AML subtypes based on CBF status could also be explained at the proteome level. The three proteins that were significantly identified among three differential strategies included *ANPEP* (also known as CD13), *MMP14*, and *AK5*. *ANPEP* (*CD13*) as a potential target in AML is being investigated in a bispecific, and split CAR-T cells targeting *CD13* and *TIM3* as *CD13* was shown to be overexpressed in AML cells [52]. *MMP14* is in the matrix metalloproteinase family and is involved in the breakdown of the extracellular matrix in normal physiological processes. MMP inhibitor (MMPI) is also being investigated in the context of AML, as MMPI reduces AML growth, prevents stem cell loss, and improves chemotherapy effectiveness [53]. Next, *AK5* is a member of adenylate kinase family that was previous shown to be highly expressed in leukemia stem cells (LSCs) compared to human hematopoietic stem cells (HSCs), indicating potential therapeutic targets for quiescent and chemotherapy-resistant human LSCs [54]. Overall, our study suggested that *ANPEP* (*CD13*), *MMP14*, and *AK5* are overexpressed in non-CBF compared to CBF AML and could be interesting drug targets. However, they are underexpressed in both MRD1-positive and Ara-C LC50-high level, suggesting further studies are needed to investigate this phenomenon.

Finally, the significant correlation between the proteome and transcriptome within 15 samples was lower than expected, implying that changes in the transcriptome only resulted in minor or transient changes in the proteome of leukemic cells. However, this could be due to the small sample size, a limitation in our pilot study.

## 5. Conclusions

In conclusion, we report the first global untargeted proteomic analysis of leukemic cells in pediatric AML, leading to the validation of biological function between CBF and non-CBF AML subtypes and new insights into AML pathobiology and potential druggable targets. This pilot study also demonstrates the high feasibility and importance of global proteomic analysis in pediatric AML. To further support these results, we are currently expanding our proteomic research in a larger cohort.

## Figures and Tables

**Table 1:** Characteristics of 16 pediatric AML patients with CBF or Non-CBF AML.

Sample ID	Age (Year)	Race	WBC (10^9^/L)	CBF Status	FLT3 Status	MRD1	Treatment Arm	Ara-C LC50 (ng/μL)
S1	3.71	White	38.9	CBF	WT	Negative	HDAC	1.18
S2	21.20	White	70.2	NON-CBF	Mutation	Negative	LDAC	NA
S3	10.29	Black	35.2	CBF	WT	Negative	HDAC	0.23
S4	6.16	White	28.7	NON-CBF	WT	Positive	HDAC	0.39
S5	12.58	White	24.3	NON-CBF	WT	Negative	HDAC	0.13
S6	15.29	White	15.0	NON-CBF	WT	Positive	LDAC	NA
S7	11.23	White	351.0	CBF	WT	Negative	HDAC	0.14
S8	4.07	White	39.9	NON-CBF	WT	Positive	LDAC	1.79
S9	11.70	Black	76.6	NON-CBF	ITD	Positive	HDAC	0.37
S10	13.05	White	34.3	NON-CBF	WT	Positive	LDAC	NA
S11	3.04	White	5.9	NON-CBF	WT	Positive	HDAC	0.70
S12	5.46	White	24.6	CBF	WT	Positive	HDAC	0.34
S13	12.69	White	247.9	NON-CBF	ITD	Positive	LDAC	0.21
S14	5.39	White	6.7	NON-CBF	WT	Negative	HDAC	0.12
S15	16.53	White	19.0	CBF	WT	Negative	LDAC	0.29
S16	11.52	White	32.0	CBF	WT	Negative	LDAC	0.01

WBC, White Blood Cells; CBF, Core Binding Factor; MRD1, Minimal Residual Disease Post Induction 1; Ara-C, Cytarabine; WT, Wild type; ITD, Internal tandem duplication.

## Data Availability

The data presented in this study are available on request from the corresponding author.

## Supplementary Materials

The following are available online at https://www.mdpi.com/article/10.3390/cancers13133161/s1, Figure S1: Leukemic cell proteomic profiles comparing MRD1 positive with negative in 16 AML patients, Figure S2: Leukemic cell proteomic profiles comparing Ara-C LC50 high with negative in 13 patients with available LC50 level, Table S1: Significant differential expressed proteins between CBF and non-CBF AML, Table S2: Significant results of GSEA (FDR < 0.05) on 9800 proteins in CBF status comparison utilizing GO, KEGG, and REACTOME databases, Table S3: Significant differential expressed proteins between MRD1 positive and negative in 16 AML patients, Table S4: Significant differential expressed proteins between Ara-C LC50 high and low in 13 patients with available LC50, and Table S5: Integrative Analysis of at least two significant differential expressed proteins among three comparator status (CBF, MRD1, and Ara-C LC50).
